# Antiproliferative Activity of Double Point Modified Analogs of 1,25-Dihydroxyvitamin D_2_ Against Human Malignant Melanoma Cell Lines

**DOI:** 10.3390/ijms17010076

**Published:** 2016-01-08

**Authors:** Anna Piotrowska, Justyna Wierzbicka, Sharmin Nadkarni, Geoffrey Brown, Andrzej Kutner, Michał A. Żmijewski

**Affiliations:** 1 Department of Histology, Faculty of Medicine, Medical University of Gdańsk, 1a Debinki, Gdańsk 80-211, Poland; annapiotrowska@gumed.edu.pl (A.P.); jwierzbicka@gumed.edu.pl (J.W.); 2 Pharmaceutical Research Institute, 8 Rydygiera, Warsaw 01-793, Poland; s.nadkarni@ifarm.eu (S.N.); a.kutner@ifarm.eu (A.K.); 3 School of Immunity and Infection, University of Birmingham, Vincent Drive, Edgbaston, Birmingham, West Midlands B15 2TT, UK; g.brown@bham.ac.uk

**Keywords:** vitamin D, vitamin D_2_, novel vitamin D analogs, melanoma, skin cancer, VDR

## Abstract

Vitamin D is a lipid soluble steroid hormone with pleiotropic biological properties, including regulation of cell proliferation, differentiation and apoptosis. As to these desirable anticancer actions, 1,25-dihydroxyvitamins D and analogs have been reported to inhibit the proliferation and to induce differentiation of a wide variety of cancer cell types, including human malignant melanoma. However, there is a need for novel and more efficacious vitamin D analogs, and how best to design such is still an open issue. A series of double point modified (DPM) analogs of 1,25-dihydroxyvitamin D_2_ (1,25(OH)_2_D_2_) induced differentiation of the vitamin D receptor (VDR) positive A375 and VDR negative SK-MEL 188b human malignant melanoma cell lines. Surprisingly, the dose of 1,25(OH)_2_D_2_ required to inhibit the proliferation of the A375 melanoma cell line by was several fold lower than that required in the case of 1,25(OH)_2_D_3_. To evaluate the impact of the modification in the side chain (additional 22-hydroxyl) and in the A-ring (5,6-trans modification), the regular side-chain of vitamin D_2_ or D_3_ was retained in the structure of our analogs. As expected, 5,6-*trans* modification was advantageous to enhancing the anti-proliferative activity of analogs, but not as a single point modification (SPM). Very unexpectedly, the additional 22-hydroxyl in the side-chain reduced significantly the anti-proliferative activity of both the natural and 5,6-*trans* series analogs. Finally, an induction of pigmentation in melanoma SK-MEL 188b cells was observed to sensitized cells to the effect of vitamin D analogs.

## 1. Introduction

**Figure 1:**
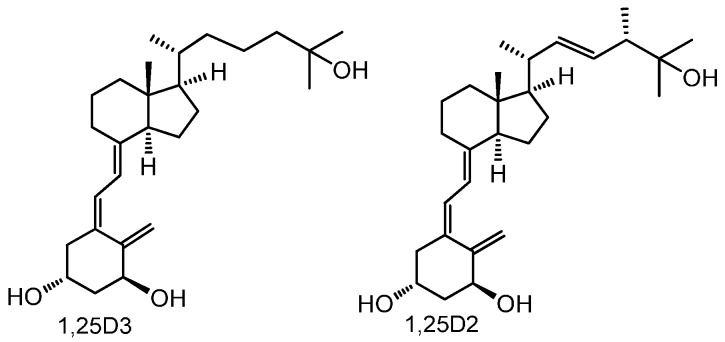
Structures of 1,25-dihydroxyvitamin D_3_ (1,25(OH)_2_D_3_) and 1,25-dihydroxyvitamin D_2_ (1,25(OH)_2_D_2_).

Vitamin D is perhaps the oldest existing hormone [1,2]. As early as 750 million years ago, phytoplankton and zooplankton produced vitamin D in the oceans [2]. There are two forms of vitamin D—ergocalciferol (D_2_) and cholecalciferol (D_3_) [3], which differ from each other in a ∆^22^ double bound and a methyl in the side chain at C-24 in vitamin D_2_ (Figure 1) [4].

Simple life forms, like krill or brine shrimp, contain 7-dehydrocholesterol (7-DHC) as well as ergosterol, the precursors for vitamin D_3_ and D_2_, respectively [1]. Vitamin D_3_ is mainly produced in the skin via photolysis of 7-DHC through a two-step process in which the B-ring is broken in a photolytic process driven by ultraviolet B (UVB) irradiation, giving the previtamin D_3_ [4]. Previtamin D_3_ very quickly isomerizes to vitamin D_3_ in a thermal non-catalytic process [4]. By contrast, vitamin D_2_ is synthesized in plants and fungi from UVB-irradiated ergosterol [4].

It is well established that vitamin D_2_ is as effective as vitamin D_3_ in maintaining the proper level of the main circulating vitamin D metabolite, which is 25-hydroxyvitamin D (25(OH)D) [5].

Vitamin D is biologically inert and regardless of the source it requires two subsequent steps of hydroxylations to gain activity. The first, at C-25, takes place in the liver and second, at C-1 in an α orientation, in the kidney [6]. The essential and the most widely known role of vitamin D is regulation of calcium homeostasis for bone health [7]. However, the activity of the hormonal form of vitamin D extends far beyond mineral homeostasis and skeletal health maintenance [3]. The widespread expression of vitamin D receptor (VDR) and the enzyme responsible for final activation of 25(OH)D tissues emphasizes the diversity of vitamin D-dependent regulatory mechanisms that are both autocrine and paracrine [1,8,9,10,11,12,13]. As to function of vitamin D, this can be elicited by genomic and non-genomic mechanisms [14]. By binding to vitamin D response elements (VDRE) in the promoter regions [15,16,17], 1,25(OH)_2_D may regulate at least 3000 genes in the human genome [14]. The non-genomic and rapid response to 1,25(OH)_2_D relies on signal transduction pathways leading to modulation of the intracellular concentration of calcium [2,14].

Regulation of calcium concentration in serum as required for proper bone mineralization is the major function of the hormonal form of vitamin D [7,18]. It should be emphasized, however, that the role of activated cholecalciferol is much more diverse. The non-calcemic effects of vitamin D include regulation of proliferation, differentiation and apoptosis [19,20,21,22,23,24], and 1,25(OH)_2_D is one of the most potent inhibitors of cell growth [25,26]. Therefore, vitamin D analogs are considered as promising anticancer compounds, which is supported by data from preclinical studies and epidemiological that relate low levels of vitamin D to an increased risk of cancer [27,28,29,30,31,32,33,34,35,36,37,38,39,40].

Previously, we have investigated the activities of low calcemic analogs of vitamin D_3_ with 20-hydroxyl, as well as analogs with a short side chain ([41,42], also see [40,43] for recent reviews). In our continuous search for more active vitamin D analogs for use as potential therapeutics [44], we have investigated the analogs of 1,25-dihydroxyvitamin D_2_ (1,25(OH)_2_D_2_) [45]. Following the discovery [46] of an ample free space around the terminal part of the side-chain we designed [47], synthesized [48] and investigated the biological activity of side-chain homologated analogs [49]. A favorable activity profile was observed for analogs containing a side chain with an ATRA-like all-*trans* geometry [50]. However, in order to evaluate the importance of double point modifications (DPM) in the vitamin D molecule, *i.e.*, in the A-ring (5,6-*trans*) and in the side-chain (22-hydroxyl and 24-*epi*), we returned our attention to analogs with the regular untouched D_2_- or D_3_-like side-chain. 

In this study, we have investigated the capacity of our series of (DPM) analogs of 1,25-dihydroxyvitamin D_2_ to inhibit cell proliferation of the human malignant melanoma VDR positive A375 and VDR depleted SK-MEL 188b cell lines.

## 2. Results

### 2.1. New Vitamin D Analogs Effectively Inhibit A375 Cell Proliferation

**Figure 2:**
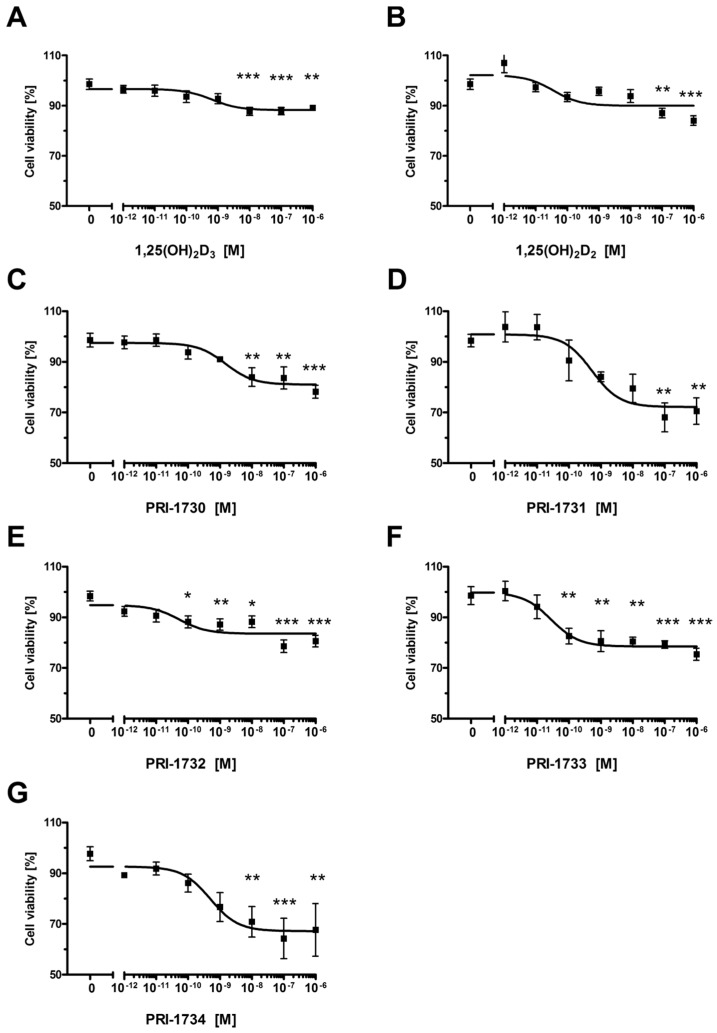
The effect of vitamin D analogs on the proliferation of human malignant melanoma A375 cells. The cells were treated with serial dilutions (10^−12^–10^−6^ M) of 1,25(OH)_2_D_3_ (**A**), 1,25(OH)_2_D_2_ (**B**), PRI-1730–PRI-1734 (panels (**C**–**G**), respectively) for 24 h. Data are shown as mean from three independent experiments ± S.D. Statistical significance was estimated using One–Way ANOVA and presented as * *p* < 0.05, ** *p* < 0.005, *** *p* < 0.0005 *vs.* control.

Figure 2 shows the effect of 1,25(OH)_2_D_3_, 1,25(OH)_2_D_2_ and the analogs PRI-1730–PRI-1734 on the proliferation of the human melanoma cell line A375. These analogs inhibited the proliferation of A375 melanoma cells, as determined using the Sulphrodamin B assay (SRB) (Figure 2A–G). IC_50_ values ranged from 1.5 nM for PRI-1730 to around 0.028 nM, with the highest activity observed for PRI-1733 (Table 1). The effect of the analogs varied as to the level of maximal inhibition of proliferation which ranged from 10%–15% for 1,25(OH)_2_D_3_ and 1,25(OH)_2_D_2_ to 20%–30% for the new analogs.

**Table 1:** Summary of the IC_50_ values for inhibition of proliferation of the human malignant melanoma A375 cells.

Compound	IC_50_ (nM)
1,25(OH)_2_D_3_	0.656
1,25(OH)_2_D_2_	0.036
PRI-1730	1.500
PRI-1731	0.524
PRI-1732	0.053
PRI-1733	0.028
PRI-1734	0.497

### 2.2. Treatment of A375 Human Malignant Melanoma Cells with the New Vitamin D Analogs Resulted in G0/G1 Arrest

**Figure 3:**
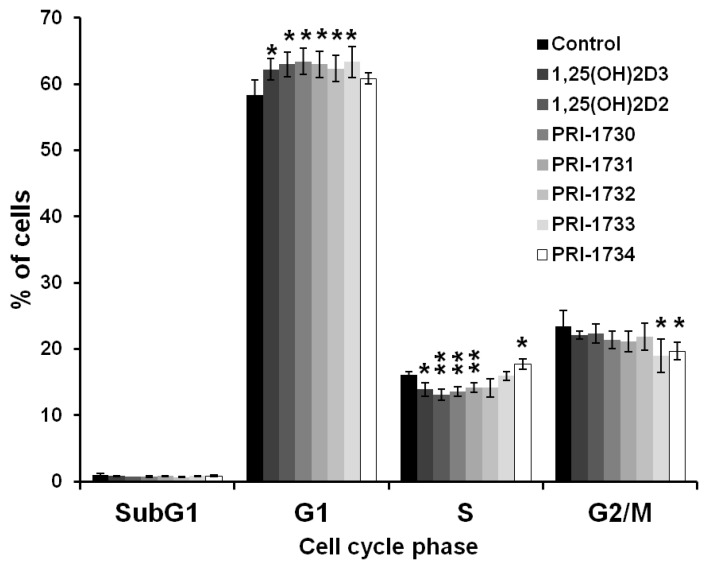
Effect of 24 h incubation with vitamin D analogs at 100 nM concentration on the distribution of human malignant melanoma A375 cells throughout phases of the cell cycle (SubG1—apoptotic/necrotic cells, G1—growth, S—DNA synthesis, G2/M—preparation for mitosis/mitosis). Cells were harvested, stained with propidium iodide and analyzed by Flow Cytometry. Data are presented as mean ± S.D. of three independent experiments carried out in triplicate. * *p* < 0.05; ** *p* < 0.01 *vs.* control.

To further investigate the inhibition of A375 cell proliferation by the new vitamin D analogs, we analyzed the extent to which analogs affected the distribution of melanoma cells in various phases of the cell cycle by flow cytometry. All the analogs tested, except PRI-1734, increased the percentage of melanoma cells in the G0/G1 phase of cell cycle and this was accompanied by proportional decrease in the number of cells in the S and G2/M phases of cell cycle (Figure 3).

### 2.3. SK-MEL 188b Melanoma Cells Do Not Express VDR Receptor and Vitamin D 24-Hydroxylase (CYP24A1)

It is well established that inhibition of proliferation and stimulation of differentiation of various cancer cell lines by vitamin D and its analogs requires the expression and activity of VDR. To establish whether the biological activity of the newly synthesized analogs of vitamin D against melanoma cells required the presence of active VDR we tested analogs for activity against the melanoma cell line SK-MEL 188b. SK-MEL 188b is a spontaneous subclone of SK-MEL 188b melanoma that lacks active VDR.

**Figure 4:**
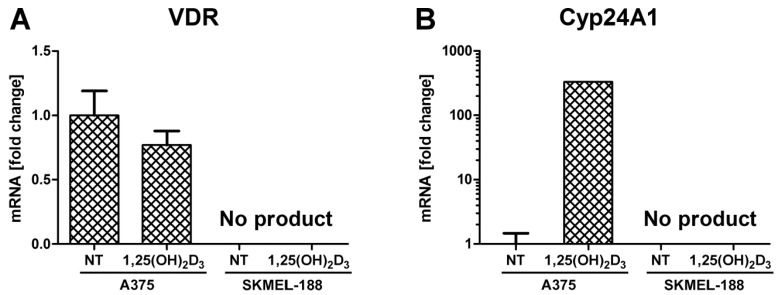
Effects of 1,25(OH)_2_D_3_ on VRD (**A**) and CYP24A1 (**B**) gene expression in A375 and SK-MEL 188b human malignant melanoma cells. mRNA levels were measured by qPCR. Data are shown as means ± S.D of three independent experiments carried out in duplicate. NT—not treated, control cells.

Post-treatment of A375 melanoma with 1,25(OH)_2_D_3_, expression of VDR was inhibited slightly, while CYP24A1, the vitamin D catabolic enzyme, was strongly induced (Figure 4). Transcripts for VDR and CYP24A1 genes were not detected in SK-MEL188b cells.

Furthermore, incubation of human malignant melanoma SK-MEL 188b cells with active form of vitamin D_3_ did not lead to the appearance mRNAs for either VDR or CYP24A1 (Figure 4).

### 2.4. Inhibition of Melanoma Proliferation by Novel Vitamin D Analogs Is Reliant on VDR

To resolve whether the effect exerted by new vitamin D analogs on A375 cells is dependent on VDR, we tested analogs for activity against SK-MEL 188b human malignant melanoma cells which, as above, lack VDR (see Figure 4).

**Figure 5:**
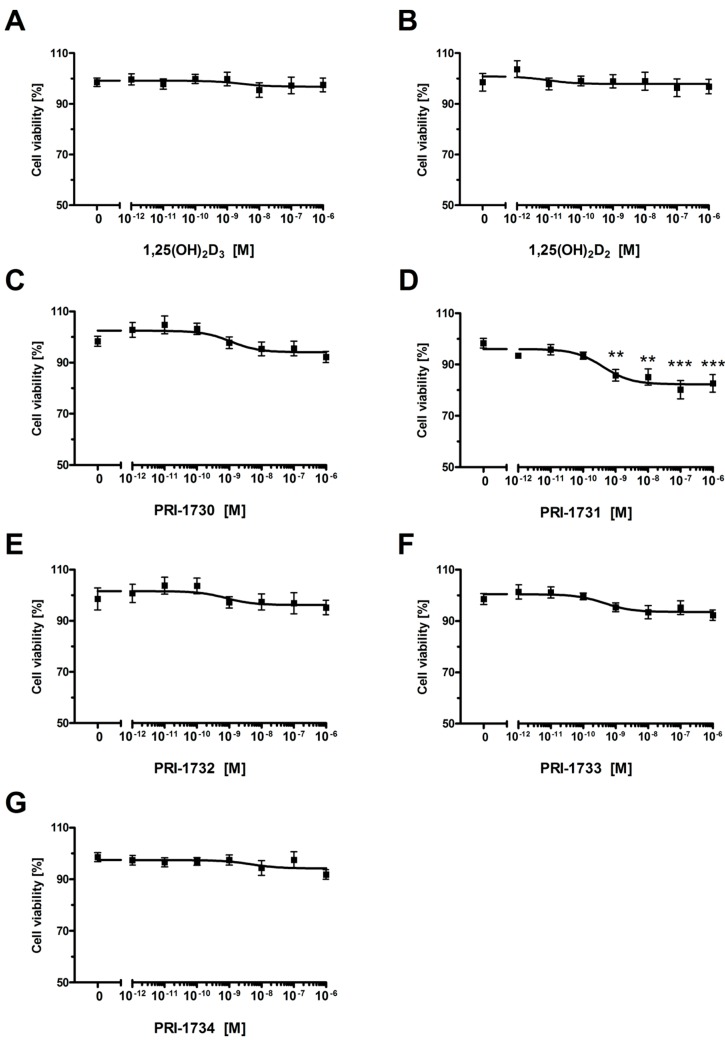
The effect of Vitamin D analogs on the proliferation of the human malignant melanoma SK-MEL 188 cells post-treatment with serial dilutions (10^−12^–10^−6^ M) of 1,25(OH)_2_D_3_ (**A**), 1,25(OH)_2_D_2_ (**B**), PRI-1730–PRI-1734 (panels (**C**–**G**), respectively) for 24 h. Data shown are the mean from three independent experiments ± S.D. Statistical significance was estimated using One–Way ANOVA and presented as ** *p* < 0.005, *** *p* < 0.0005 *vs.* control.

The new vitamin D analogs had only a very minor influence on non-pigmented SK-MEL 188b melanoma cells (Figure 5). The levels of inhibition observed were not statistically significant for all compounds other than PRI-1631, which gave an IC_50_ value of 0.408 nM. We were not able to calculate valid IC_50_ values for the remaining compounds (Table 2). Furthermore, at most we only observed approximately a 10% decrease in cell viability even at the maximal concentration used of 1 μM. The PRI-1731 analog was the only analog that decreased viability of SK-MEL 188b melanoma cells to a level of approximately 20% (Figure 5D).

**Table 2:** Summary of IC_50_ values for inhibition of proliferation of non-pigmented human malignant melanoma SK-MEL 188 cells. NS—not significant.

Compound	IC_50_ (nM)
1,25(OH)_2_D_3_	NS
1,25(OH)_2_D_2_	NS
PRI-1730	NS
PRI-1731	0.408
PRI-1732	NS
PRI-1733	NS
PRI-1734	NS

### 2.5. New Vitamin D Analogs Had Only a Very Limited Effect on Non-Pigmented SK-MEL 188b Melanoma Cells as to the Distribution of Cells in Phases of the Cell Cycle. NS—Not Significant

**Figure 6:**
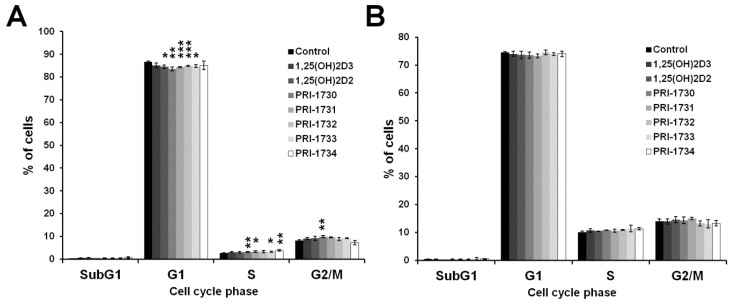
Effect of 24 h incubation with vitamin D analogs at 100 nM (**A**) and 1000 nM (**B**) concentration on the distribution of non-pigmented human malignant melanoma SK-MEL 188 cells throughout the phases of cell cycle. Cells were harvested, stained with propidium iodide and analyzed by Flow Cytometry. Data are the mean ± S.D. of three independent experiments carried out in triplicate. * *p* < 0.05; ** *p* < 0.01; *** *p* < 0.001 *vs.* control.

Figure 6 shows that the new vitamin D analogs had very little effect on the distribution of SK-MEL 188b melanoma cells in various phases of the cell cycle. In fact, vitamin D analogs at 100 nM concentration increased the percentage of melanoma cells in S and G2/M and there was a decrease in number of cells in G1 (Figure 6A). This effect is opposite to that provoked by vitamin D analogs in the case of A375 cells, perhaps due to different genetic background (a lack of VDR in SK-MEL 188b) and an activation of alternative pathways (e.g., PDIA-3 rapid response [2,14]). At higher concentrations of analogs (1000 nM) the observed effects against SK-MEL 1288 cells were not statistically significant (Figure 6B).

### 2.6. Pigmentation of SK-MEL 188 Cells Sensitizes Them to the Effects of the New Vitamin D Analogs

SK-MEL188 melanoma cells grown in F10 medium did not produce visible pigmentation, but changing in concentration of tyrosine in medium from 11 to 217 μM stimulated pigmentation. This effect was obtained by mixing DMEM and F10 (50:50, *v*/*v*).

Previous studies have indicated that pigmentation can impair both the metabolism and activity of classical vitamin D derivatives in human melanomas [51,52,53]. However, in the case of some vitamin analogs, such as 21(OH)pD [54], pigment producing SKMEL 188 cells are more sensitive to treatment. Therefore, we evaluated the responsiveness of pigmented SK-MEL 188b cells (subclone of SK-MEL 188 with no VDR expression) to new vitamin D analogs.

**Figure 7:**
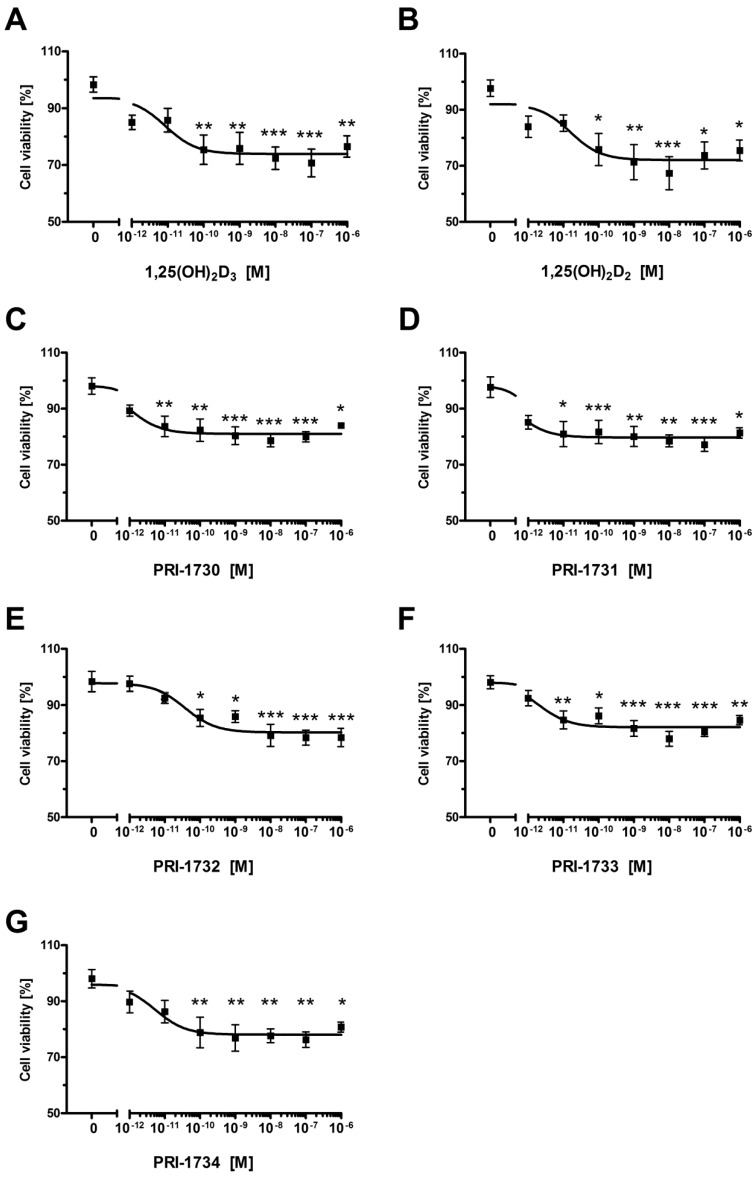
The effect of Vitamin D analogs on the proliferation of pigmented human malignant melanoma SK-MEL 188b cells. The cells were treated with serial dilutions (10^−12^–10^−6^ M) of 1,25(OH)_2_D_3_ (**A**), 1,25(OH)_2_D_2_ (**B**), PRI-1730—PRI-1734 (panels (**C**–**G**), respectively) for 24 h. Data shown are the mean from three independent experiments ± S.D. Statistical significance was estimated using One–Way ANOVA and presented as * *p* < 0.05, ** *p* < 0.005, *** *p* < 0.0005 *vs.* control.

Interestingly, the pigmented SK-MEL188b cells were significantly sensitized to the new vitamin D analogs (Figure 7) as compared with non-pigmented cells (Figure 5). IC_50_ values ranged from 0.04 nM for PRI-1732 to approximately 0.0004 nM, with the strongest effect being seen for PRI-1731 (Table 3).

**Table 3:** Summary of IC_50_ values for the inhibition of proliferation of pigmented human malignant melanoma SK-MEL 188 cells.

Compound	IC_50_ (nM)
1,25(OH)_2_D_3_	0.0087
1,25(OH)_2_D_2_	0.0158
PRI-1730	0.0011
PRI-1731	0.0004
PRI-1732	0.0380
PRI-1733	0.0020
PRI-1734	0.0054

### 2.7. Pigmentation of SK-MEL 188b Cells Affected Cell Cycle Distribution of Melanoma Cells after Treatment with Vitamin D Analogs

**Figure 8:**
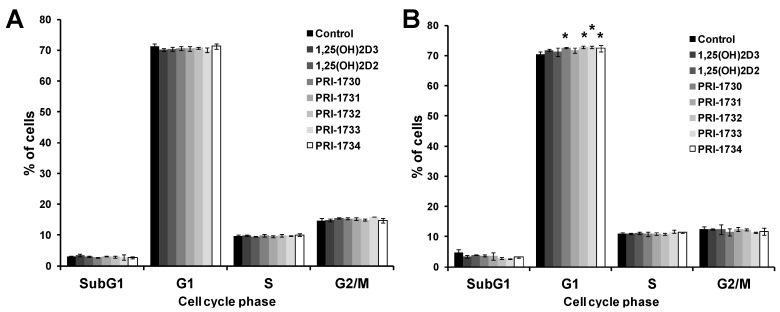
Effect of 24 h incubation with vitamin D analogs at 100 nM (**A**) and 1000 nM (**B**) concentration on the distribution of pigmented human malignant melanoma SK-MEL 188 cells throughout the phases of cell cycle. Cells were harvested, stained with propidium iodide and analyzed by Flow Cytometry. Data presented are the mean ± S.D. of three independent experiments carried out in triplicate. * *p* < 0.05 *vs.* control.

When analogs were tested at 100 nM against pigmented SK-MEL 188b melanoma cells there was no effect on the distribution of cells in the various phases of cell cycle (Figure 8A). Increasing the concentration of vitamin D analogs to 1000 nM resulted in an effect similar to that observed previously for A375 cells, as to an increase in the percentage of melanoma cells in the G0/G1 phases of cell cycle (Figure 8B).

## 3. Discussion

There is good evidence from experimental studies to support the development of vitamins D as an anticancer therapeutic [40,55]. In keeping, there is a strong correlation between an adequate level of 25(OH)D and a decreased incidence of many different malignancies, including melanoma [35,36,37,38,39,56]. However, there is a need to develop more effective vitamin D analogs.

**Figure 9:**
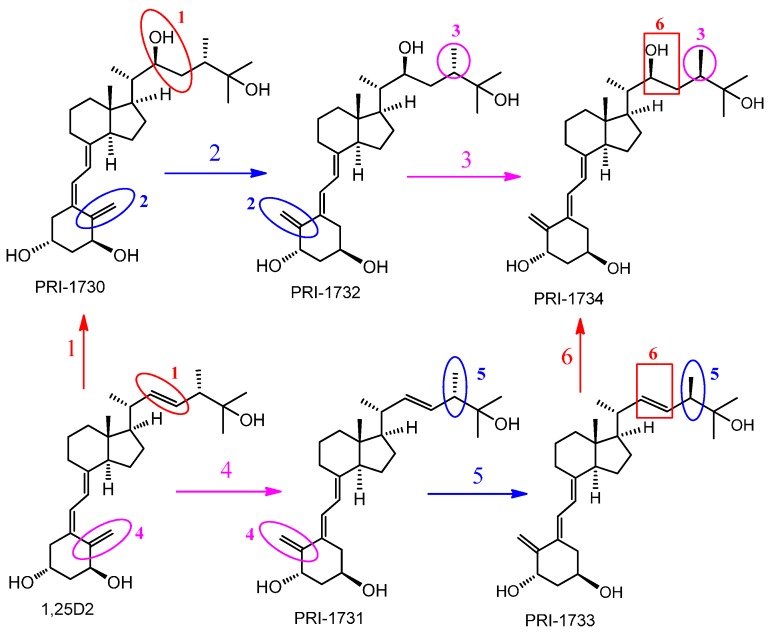
Structures of analogs of 1,25-dihydroxyvitamin D_2_ (1,25(OH)_2_D_2_) and the modifications introduced. 1 and 6—introducing 22-hydroxyl and saturation in the side-chain, 2 and 4—introducing (5*Z*,7*Z*) modification, 3 and 5—inversion of the absolute configuration at C-24.

Incubation of A375 melanoma cells with 1,25(OH)_2_D_2_ and 1,25(OH)_2_D_3_ for 24 h resulted in around a 10% decrease in the viability of cells (Figure 2). This inhibitory effect of 1,25(OH)_2_D_3_ on proliferation is consistent with previous reports [40,43,57]. Interestingly, human malignant melanoma A375 cells showed an increased sensitivity to our DPM analogs of 1,25(OH)_2_D_2_ as to inhibit proliferation of these cells required several times less 1,25(OH)_2_D_2_ than the amount of 1,25(OH)_2_D_3_ required. Quite unexpectedly, introducing 22-hydroxyl and saturation in the side-chain (Figure 9, modification **1**) of 1,25(OH)_2_D_2_ led to a reduced activity as to the resulting analog PRI-1730 (Table 1, IC_50_ 1.500 nM compared to 0.036 for 1,25(OH)_2_D_2_). A likely explanation is the addition of 22-hydroxyl, as the ∆^22^ unsaturation in the side-chain, has long been known [58] to not exert a substantial influence on activity. On the contrary, introducing 5,6-*trans*, *i.e.*, (5*Z*,7*Z*) modification **2** into the structure of PRI-1730 gave the analog PRI-1732 that inhibited proliferation of A375 cells at 30-fold lower concentrations (IC_50_ 0.053 compared to 1.500 for PRI-1730). However, this modification (**4**) was not effective when introduced as a single point modification (SPM) [59] in the structure of plain 1,25(OH)_2_D_2_ and led to a 15-fold reduction of the activity in the resulting analog. Further inversion of the absolute configuration at C-24 in PRI-1732 (modification **3**) reduced the activity of the analog PRI-1734 10-fold (IC_50_ 0.497 for PRI-1734 compared to 0.053 for PRI-1732). Interestingly, the same inversion (modification **5**) in PRI-1731 into PRI-1733 increased the activity, (from an IC_50_ value of 0.524 to 0.028) indicating 5,6-*trans* modification exerts a dominating effect when the activity reducing 22-hydroxyl is absent. Finally, introducing 22-hydroxyl (modification **6**) into the structure of 24-*epi*-5,6-*trans* analog PRI-1733 also reduced the activity, a in the case of modification **1**. PRI-1734 was slightly more active than 1,25(OH)_2_D_3_ (IC_50_ for PRI-1734 0.497 and for 1,25(OH)_2_D_3_ 0.656) and this effect appears to be specific for melanoma cells and PRI-1734 is inactive against the human promyelocytic leukemia cell line HL-60 [60].

The effect exerted on cell proliferation by our novel analogs was observed to be dependent on the presence of VDR in cells, as the SK-MEL 188b cells which lack VDR expression were largely unresponsive to the analogs Indeed, a lack of VDR has been reported to be a mechanism that underlies unresponsiveness of melanoma cells to the antiproliferative effects of vitamin D analogs, as reported by Seifert *et al.* in the case of the SK-MEL 5 cell line [61] (see also Szyszka *et al.* [40] for recent review). On the other hand, our data have shown that moderate pigmentation of SK-MEL 188b sensitizes them to the effect of vitamin D analogs (Figure 7). A similar finding has been reported from studies of murine melanoma cell lines [41], in which pigmentation increased the expression of VDR and CYP24A1 at the mRNA level, and of SK-MEL 188 human melanoma cells [54]. Interestingly, the analog PRI-1731 was the only one that significantly inhibited the proliferation of non-pigmented SK-MEL 188b melanoma cells. This analog exerted also the strongest effect against pigmented SK-MEL 188b cells, having an IC_50_ value of 0.0004 nM (Table 3).

Finally, it is important to emphasize that the new vitamin D_2_ analogs maybe an effective alternative to 1,25(OH)_2_D_3_ in the treatment of melanoma. Furthermore, the anti-proliferative activity of compounds investigated against pigmented melanoma cells is of special interest because pigmentation is usually associated with an enhanced drug resistance.

## 4. Materials and Methods

### 4.1. Vitamin D Analogs

1,25-Dihydroxyvitamin D_2_ and the analogs PRI-1730, PRI-1731, PRI-1732, PRI-1733 and PRI-1734 (Figure 9) were synthesized [62] at the Chemistry Department of the Pharmaceutical Research Institute, Warsaw, Poland. The compounds gave analytical data (^1^H and ^13^C NMR spectra, recorded on a Varian GEMINI-200, Varian S 500 and Varian S 600 spectrometers, Varian Medical Systems, Inc., Palo Alto, CA, USA; UV spectra, taken in ethanolic solutions on a Shimadzu UV-160A spectrophotometer, Shimadzu Corp., Kyoto, Japan; mass spectra (MS) and high-resolution MS (HRMS), recorded on a Maldi Spectrometer SYNAPT G2-S HDMS; Waters Corp., Milford, MA, USA) consistent with the assigned structures. Amber glass ampoules were filled with ethanolic solution of 50 μg of analogs and the solutions were dried down under a stream of argon and the ampoules flame sealed. The quantity of an analog in an ampoule was confirmed by UV.

### 4.2. Cell Culture

Human malignant melanoma A375 cells and SK-MEL 188b (a spontaneous sub-clone of SK-MEL188 that does not expressing VDR) cells were cultured in DMEM (Sigma, Poznan, Poland) and Ham’s F-10 media (Sigma), respectively, supplemented with 10% fetal bovine serum (Sigma) and 1% penicillin/streptomycin (Sigma). Charcoal-stripped fetal bovine serum was used in all experiments to examine the effects of vitamin D analogs.

Culturing SK-MEL 188b melanoma cells in a medium high in tyrosine induced a rapid production melanin [63] with attendant changes in the differentiation status of cells. The tyrosine concentration in the medium used, 50:50 DMEM:F-10, was 217 μM.

### 4.3. Proliferation Assay

To measure the effects of analogs on cell proliferation, cells were seeded in 96 well plates at a density 5000 cells per well and after 24 h were treated with serial dilutions of the compounds being tested for an additional 24 h. Following incubation cells were fixed with 10% trichloroacetic acid (TCA, Sigma) for 1 h at 4 °C. Plates were washed five times with distilled water and air-dried. A staining solution comprising 0.4% SRB (sulphorhodamine B, Sigma) in acetic acid was added to each well and after 15 min. plates were washed with 1% acetic acid five times and air-dried. SRB dye was solubilized using a solution of 10 mM buffered Tris Base (pH 10.5). Absorbance was measured at 570 nm using an Epoch spectrophotometer (BioTek, Winooski, VT, USA).

### 4.4. Cell Cycle Analysis

The cell cycle status of treated cells was analyzed by quantification of DNA content. Cells were seeded in 6 well plates at a density 150,000 cells per well and after 24 h were treated with vitamin D analogs at 100–1000 nM concentration for an additional 24 h. After fixation of cells in 70% ethanol for 24–48 h at 4 °C, cells were treated with ribonuclease in order to removed RNA contamination and DNA was stained with propidium iodide (PI, Sigma). Fluorescence of the PI-stained cells was measured by flow cytometry (Ex 536 nm, Em 617 nm, FACSCalibur, Becton Dickinson, Franklin Lakes, NJ, USA). Results were analyzed by CellQuest Pro Software (Becton Dickinson) and expressed as a percentage of cells with a DNA content corresponding to apoptotic/necrotic cells (subG1 fraction) or cells in G1, S and G2/M phases of the cycle.

### 4.5. cDNA Preparation and PCR Assays

Total RNA was isolated by using the Total RNA MiniPLUS kit (A&A Biotechnology, Gdynia, Poland), following the manufacturer’s instructions. The RNA concentrations were determined using Epoch Microplate Spectrophotometer (BioTek, Winooski, VT, USA). RNA extracted was reverse transcribed and cDNA synthesized using RevertAid™ First Strand cDNA Synthesis Kit (Thermo Fisher Scientific Inc., Waltham, MA, USA). Real Time PCR was performed using an StepOnePlus™ Real-Time PCR System (Life Technologies-Applied Biosystems, Grand Island, NY, USA) with Real Time HS 2x PCR Master Mix SYBR^®^ kit (A&A Biotechnology). The primer sets (Table 4) used for PCR were designed by authors and have been published previously [42]. RPL-37A was used as a housekeeping gene. All primers were purchased from Sigma-Aldrich, Munich, Germany. The expression of the genes were normalized by comparative ΔΔ-*C*_t_ method, using RPL37A as a housekeeping gene, followed by calibration (fold change) to normalized expression data of samples from control (ratio = 1). To ensure specificity of the PCR amplification, dynamic melting curve analysis was performed for all reactions.

**Table 4:** PCR primers data.

Gene Name	Forward Primer	Reverse Primer
*RPL37A*	TTCTGATGGCGGACTTTACC	CACTTGCTCTTTCTGTGGCA
*VDR*	CCAGTTCGTGTGAATGATGG	GTCGTCCATGGTGAAGGA
*CYP24A1*	GCAGCCTAGTGCAGATTT	ATTCACCCAGAACTGTTG

### 4.6. Statistical Analyses

Statistical analysis was performed using Microsoft Excel or GraphPad Prism v 6.03 (GraphPad Software, San Diego, CA, USA). Data were subjected to Student’s *t*-test (for two groups) or one-way analysis of variance and appropriate post hoc test (the ANOVA Kruskal–Wallis test for comparison of several groups). Data are expressed as mean ± S.D. Each experiment was repeated at least three times in triplicate. Differences are shown as significant at *p* < 0.05, *p* < 0.01 or *p* < 0.001, as indicated.

## 5. Conclusions

Many studies have reported that the biological activities of 1,25(OH)_2_D_2_ and 1,25(OH)_2_D_3_ are very similar [45]. By contrast, the human malignant melanoma cell lines A375 and SK-MEL 188b, investigated in this study responded differently to the treatment with 1,25(OH)_2_D_3_ and 1,25(OH)_2_D_2_. The response of SK-MEL 188b cells to a given analog was mostly similar to that of A375 cell line. As expected, 5,6-*trans* modification was found to be advantageous to activity, but not as a single point modification, as in the case of the formal conversion of 1,25(OH)_2_D_2_ into the analog PRI-1731.The influence of adding 22-hydroxyl in the side-chain was first evaluated. Surprisingly, this modification significantly reduced the activity of analogs as to both the natural and 5,6-*trans* series. As such, this modification might be useful when designing a vitamin D antagonist. The activities of analogs of 1,25(OH)_2_D_2_ were variable as to the cell line tested. PRI-1734 showed very low activity, as compared to 1,25(OH)_2_D_2_, when tested against A375 cells and SK-MEL 188b cells [60]. However, PRI-1734 it was still more active than 1,25(OH)_2_D_3_ against A375 cells.

The IC_50_ obtained for 1,25(OH)_2_D_2_ for inhibition of proliferation of A375 cells and SK-MEL 188b was several fold lower than that of 1,25(OH)_2_D_3_. This supports the viewpoint that analogs of 1,25(OH)_2_D_2_ may be a good alternative to 1,25(OH)_2_D_3_ and its analogs as to developing anticancer therapeutics. In this regard, it will be important to investigate whether the substantial differences observed for the activities of 1,25(OH)_2_D_2_ and 1,25(OH)_2_D_3_ against the human malignant melanoma cell lines A375 and SK-MEL 188b applies to other malignant cell lines.

## Abbreviations

1,25(OH)_2_D_2_—1,25-dihydroxyvitamin D_2_; 1,25(OH)_2_D_3_—1,25-dihydroxyvitamin D_3_ (calcitriol); 25(OH)D—25-hydroxyvitamin D; 7-DHC—7-dehydrocholesterol (provitamin D_3_, cholesta-5,7-dien-3β-ol); ATRA—All-*trans* retinoic acid; D_2_—ergocalciferol; D_3_—cholecalciferol; DPM—double point modified (analogs); HRMS—high-resolution mass spectrometry; M—mole, unit; NMR—nuclear magnetic resonance; ns—not significant; NT—not treated; SPM—single point modified (analogs); UVA/B—ultraviolet radiation A and B; VDR—vitamin D receptor; VDRE—vitamin D response elements.
